# Strengthening global health security – lessons learned from public health England’s international health regulations strengthening project

**DOI:** 10.1186/s12992-021-00794-1

**Published:** 2022-02-21

**Authors:** Cindy Carlson, Tim Shorten, Asma Khalid, Matthew Cooper, Ruth Sherratt, Giovanna Voltolina

**Affiliations:** Itad, Preece House, Davigdor Road, Hove, BN3 1RE UK

**Keywords:** International health regulations, Strengthening global health security, Health emergencies, Health systems, Evaluation

## Abstract

**Background:**

Country experiences of responding to the challenges of COVID-19 in 2020 highlighted how critical it is to have strong, in-country health security capacity. The UK government has invested in health security capacity development through various projects and agencies, including the UK Department of Health and Social Care, whose Global Health Security Programme provides funding to Public Health England (PHE) to implement health security support. This article describes the results and conclusions of the midterm evaluation, undertaken by Itad, of one of Public Health England’s global health projects: International Health Regulations Strengthening, which operates across six countries and works with the Africa Centres for Disease Control. It also highlights some of the key lessons learned for the benefit of other agencies moving into supporting national health security efforts.

**Results:**

The Itad team found strong evidence that the IHR Project is well aligned with, and responding to, partners’ capability strengthening needs and that the three workstreams – systems coordination, workforce development and technical systems strengthening are implementing relevant and appropriate action to support national priorities. The IHR Project is also aligned with and complementary to other relevant UK development assistance although the Project could strengthen the strategic collaboration with WHO, US CDC and other UK government projects in countries. The Itad team also found that the IHR Project could be more effective if the technical assistance activities were accompanied by relevant materials and equipment while maintaining its supportive role. There was evidence of where technical assistance in the form of training and follow-up mentoring had led to improvements in practice and in IHR compliance, but these were not being systematically captured by the Project’s routine reporting.

**Conclusions:**

There was good evidence that the project was doing the right things and aligning its work in the right way, with more limited evidence at the time of the midterm evaluation that it was making progress towards achieving the right results.

## Background

The onset of the COVID-19 pandemic in 2020 made global health security dominate public health attention. Every country’s COVID-19 response has stress-tested their health systems, especially their health security systems, and too many have been found wanting [1–3]. Towards the tail end of the first SARS epidemic in 2005 the World Health Organisation revised their overarching legal framework for handling public health emergencies, known as the International Health Regulations (IHR) [4]. The IHR are an international legal instrument that 196 countries have agreed to uphold. The 2020/21 COVID-19 experience revealed that the global community needs to do much more to ensure that countries are better able to prepare for, prevent, monitor, respond to and recover from any future public health emergencies [5].

The UK Department of Health and Social Care’s (DHSC’s) IHR Strengthening Project,^1^ delivered by PHE as part of the DHSC’s Global Health Security (GHS) Programme has a triple mandate: 1. Building technical capability for compliance with IHR; 2. Strengthening public health leadership; and 3. Developing sustainable public health systems. The IHR Project aims to strengthen health systems in six countries (Nigeria, Ethiopia, Sierra Leone, Myanmar, Pakistan and Zambia) and also collaborates with the Africa Centres for Disease Control (ACDC) and the WHO Africa Regional Office (AFRO) to improve global health security. PHE has been allocated £16 million of UK Official Development Assistance (ODA) over a five-year period (March 2016–March 2021) for the IHR Project [6].

The PHE IHR Project is primarily a technical assistance project, drawing on PHE specialist in-country expertise as well as the expertise within the different PHE divisions in the UK. The Project partners are national and regional entities that have a mandate to protect public health and support implementation of national commitments^2^ as signatories to the IHR. These are primarily National Public Health Institutes (NPHIs) or National Institutes of Health. Where a national public health institute has not been established, the project works with the Ministry of Health entity that has responsibility for compliance with the IHR (Myanmar and Sierra Leone).

Complementing PHE’s own system for monitoring and evaluating (M&E) its activities, Itad was contracted to provide third-party monitoring and evaluation (TPM&E) to serve three main functions: as independent monitor, as evaluator and as learning partner. Itad has a formative learning role as well as a summative accountability role, working closely and symbiotically with the IHR monitoring and evaluation system. The Itad contract has been for three years, with the team responsible for conducting midterm (2019–20) and endline evaluations (2021) together with providing M&E advice to the PHE IHR team.

The Itad TPM&E for the PHE IHR Project started in 2019 with the preparation for and implementation of a midterm evaluation of Project activities up to that point in time. This paper is a summary of the evaluation results and lessons learned at the midterm, with the aim of providing advice (primarily to the PHE project team) on enhancing the IHR Project effectiveness rather than to develop an original analytical or theoretical argument about how to undertake IHR strengthening. However, a secondary goal is to add to international learning on how to provide more effective support to countries to improve their health security capacities. Lessons learned are presented in the discussion and conclusions section.

## Methods

### Data collection

The midterm evaluation (MTE) covered the main areas of the IHR Project’s activity, focusing on its interventions across countries and regions. A mixed methods approach was used. 406 documents were reviewed, including all Project reports that were available up to the end of October 2019; UK government GHS policy and strategy documents, GHS-related progress reports; international GHS literature including other GHS project evaluations^3^ and individual country reports for the six PHE IHR countries.^4^ For non-PHE documents the review team searched Google, PubMed and individual organisation websites (e.g. UK DFID, WHO, United States Centres for Disease Control and Prevention-US CDC) using keywords: ‘global health security’, ‘International Health Regulations’, ‘health emergencies’, ‘health systems’ and ‘evaluation’.

102 key informant interviews were conducted with stakeholders including IHR Project managers and technical specialists, in-country public health institute partners, in-country UK government officials, and other development partners who were also working with these same institutions. Table 1 provides an overview of key informants that were jointly identified by the evaluation team and the IHR Project Team. These interviews were based on a standard semi-structured interview guide, tailored to the key informant being interviewed and how they engaged with the project. Most interviews were recorded, and detailed notes were taken during the interviews and then finalised by referring back to the recordings.

**Table 1:** Overview of key informants interviewed

Stakeholder group	Detail
PHE internal stakeholders	• PHE Headquarters staff, including the Director of Global Health and Director of Strategy • Global Public Health IHR team
Global and regional stakeholders	• Africa CDC • The International Association of National Public Health Institutes • Fleming Fund management agent, regional coordinators and relevant implementing partners
Country-level stakeholders	**Government stakeholders** • Representatives from national public health institutions • Ministry of Health officials • Members of AMR country coordinating staff • Ministry of Planning and Environment officials • National laboratory staff **Other country-level stakeholders** • Implementing partners such as WHO • CDC, DFID, other HMG (e.g. UK military, FCO, deployed UK Public Health Rapid Support Team staff) and other development partner staff. Endline stakeholders should also include country grantees, Fleming fellows and other implementers where appropriate. • Recipients of initiatives • Public health workforce • Those trained under the IHR project

Four of the six PHE IHR countries were visited by MTE team members (Ethiopia, Nigeria, Pakistan and Sierra Leone), as well as the Africa CDC offices in Addis Ababa, Ethiopia. Stakeholders in Myanmar and Zambia were interviewed remotely, as it was found that the Project was in too early a stage to warrant country visits to these countries at midterm. All data from the country- and Africa CDC- specific document reviews and interviews were compiled into a contribution story template that provided an overview of the Project’s focus in each country including inputs, processes and outputs.

Finally, the Itad team reviewed all the data available on the project’s task management software (Jira) and the project’s financial data, where available.

**Fig. 1:**
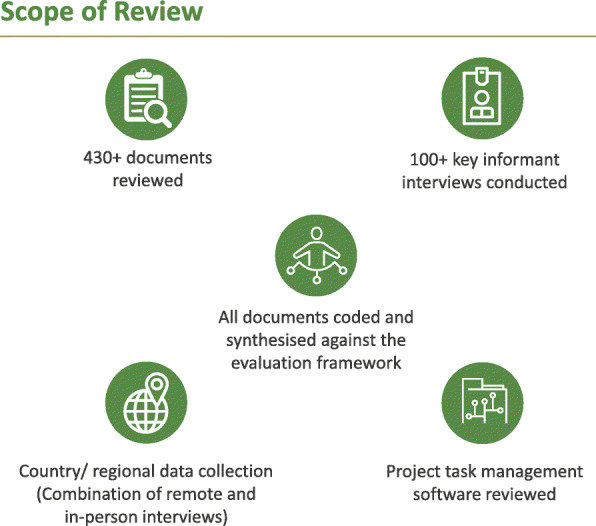
Scope of review (At country/regional-level interviews were conducted with 64 stakeholders: Nigeria (8 stakeholders), Ethiopia (15 stakeholders), Africa CDC (6 stakeholders), Myanmar (2 stakeholders), Pakistan (16 stakeholders), Sierra Leone (13 stakeholders), and Zambia (4 stakeholders).)

Figure 1 illustrates the scope of the midterm evaluation review.

### Data analysis

**Fig. 2:**
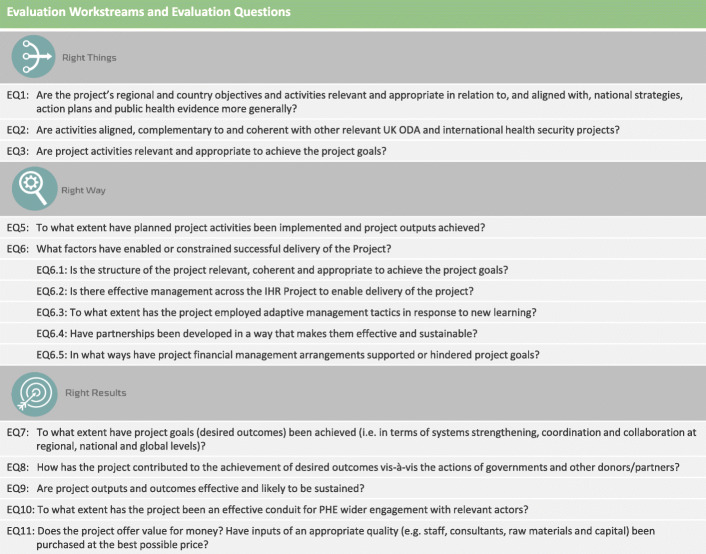
Evaluation workstreams and questions

The evaluation Terms of Reference set out a number of evaluation requirements that the team translated into evaluation questions. The organising principle underpinning the data analysis was based on whether the project was: doing the right things, doing the right things in the right way, and whether it was getting the right results. Figure 2 provides the breakdown of evaluation questions into these three categories.

Background documents and interview notes were reviewed and had excerpts coded against these evaluation questions and also against the appropriate country or regional case study using a Microsoft Excel-based coding framework. Where an excerpt was relevant to more than one evaluation question and/or case study, the excerpt was coded against all relevant codes, to ensure that the analysis against the different workstreams factored in all relevant data. All excerpts were accompanied by the title of the background document or the interview note’s unique identifier code, which supported later strength of evidence assessments.

**Fig. 3:**
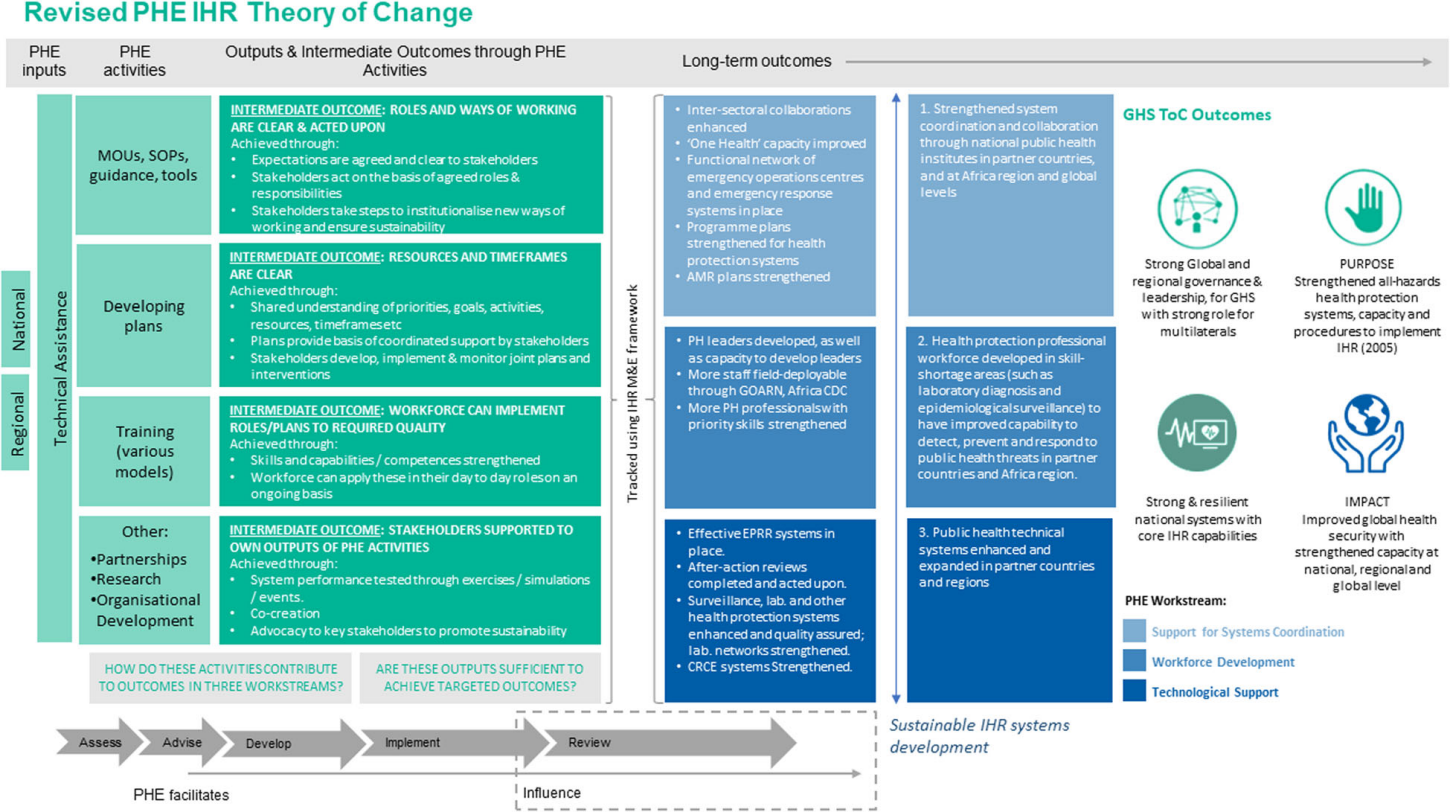
PHE IHR Project Theory of Change

The Itad team used a draft theory of change (TOC)^5^ (Fig. 3) developed with IHR Project team members as an analysis framework in the following ways: 1. to understand and assess whether assumptions that underpin the intervention logic have held in the implementation of activities, and whether there are explanatory contextual factors at play, and 2. as the basis for identifying evidence as a benchmarking tool for the relevance and appropriateness of interventions. At the start of our evaluation inception phase, the IHR Project TOC and logical framework was based on the Phalkey model, which suggests that there are three interlocking support functions – health system support, workforce support and technical/technological support – to form the basis for categorising inputs to health protection system strengthening programmes, and understanding their impact mechanism.

The Itad team undertook a contribution-informed analysis,^6^ for each country and Africa CDC, to examine PHE’s plausible contribution to country and Africa CDC IHR compliance efforts. These were then used for cross-case analysis.

As the IHR Project featured a relatively new way of working internationally for PHE, and the Itad team were evaluating whether the project is being implemented in the ‘right way’, a responsibility, accountability, consulted and informed (RACI) analysis^7^ was conducted through the key informant interviews with UK and country-based teams to better understand the various PHE IHR team members’ roles and responsibilities across project management and delivery. For each PHE process delivered/actioned by various stakeholders, the RACI analysis plotted: who is responsible, who is accountable, who is consulted and who is informed. Processes related to project design, implementation, M&E and communications functions were included. The Itad team also compared what was planned with what actually happened, over the course of the evaluation period, in order to identify areas where stakeholders are not fulfilling (or are overreaching) their expected functions, and any implications for project management and governance.

Based on the data collected from across the evaluation workstreams and questions, Itad conducted a value for money analysis. This utilised the 3Es framework developed by the UK National Audit Office (NAO) and Department for International Development (DFID) to determine:

- **Economy:** Whether inputs (e.g., staff, consultants, raw materials and capital) of an appropriate quality have been procured at the best possible price.
- **Efficiency:** The extent to which outputs were maximised for a given level of input.
- **Effectiveness:** How well outputs achieved/delivered desired outcomes.

The results of this analysis were communicated through a rubric designed to provide a transparent ranking of performance based on clear definitions of what poor, adequate, good and excellent value for money looks like for each area.

As the evaluation is informed by a utility-focused evaluation^8^ approach, Itad remained focused throughout the evaluation on using existing as well as generating new evidence. Once data collection was completed, the evaluation team held a two-day workshop to develop an agreed analysis on ‘right things’, ‘right way’ and ‘right results’. The preliminary results, analysis and recommendations were then shared and discussed with key members of the PHE team at a co-creation workshop, while the MTE report was being drafted.

## Results

### Is the IHR project doing the right things?

In analysing whether the project was ‘doing the right things’, the Itad team considered the alignment of the IHR Project’s actions with national need and global standards, as well as the relevance of these actions for achieving the project goals.

The Itad team found strong evidence that the IHR Project is well aligned with, and responding to, partners’ capability strengthening needs as defined by national IHR Joint External Evaluation (JEE) assessments^9^. The PHE IHR Project team undertook a number of scoping missions to countries before developing interventions that were tailored to the needs reflected in JEE reports, national requests for assistance, and also taking into account what other GHS stakeholders were doing and PHE’s own areas of expertise. The results of these scoping missions were written up in considerable detail, which included what were at the time the latest JEE results and national government stakeholders prioritised requests for PHE IHR strengthening assistance. Table 2 provides some illustrative examples from Nigeria and Myanmar.

**Table 2:** Illustrative examples of IHR Project alignment with country IHR priorities

**Nigeria**: In Nigeria, the country’s capacity on biosafety and security was one area that needed to be strengthened, according to the 2017 JEE. At the point of the MTE, PHE had run two sets of training on biosafety in laboratories, including for Lassa Fever, and supported an audit of biosafety requirements. PHE had further supported the implementation of recommendations from these exercises.	
**Myanmar:** The IHR Project’s partnership with the Myanmar National Poisons Control Centre (NPCC) on strengthening national poisons and chemicals management capability and capacity resulted in 41 healthcare staff, based at Mandalay and Magway General Hospitals, undertaking essential TOXBASE® e-learning. In addition, the Project’s engagement activities promoted enhanced co-creation and collaboration with national stakeholders on poisons and chemicals management, including WHO Myanmar office, Occupational and Environmental Health Division from the University of Public Health, NPCC and New Yangon General Hospital, Mandalay and Magway General Hospitals.	

A mentioned above, the IHR Project was designed to implement three concurrent workstreams – systems coordination, workforce development and technical systems strengthening. These workstreams have evolved over the course of the Project to respond to the Project’s triple mandate: building IHR related technical capability, strengthening public health leadership and developing sustainable public health systems. Each workstream was found to be implementing relevant and appropriate action to support national priorities.

The IHR Project is also aligned with and complementary to other relevant UK development assistance^10^ and other global health security projects, for example, those delivered by the World Health Organisation (WHO) and the US Centres for Disease Control and Prevention (US CDC). The PHE teams made a substantial effort to ensure that the IHR Project activities did not duplicate or contradict the work of other organisations in the six countries and in Africa CDC. However, there were areas where the evaluation team felt the project could strengthen the strategic collaboration with WHO, US CDC and other UK government projects in countries, while acknowledging that such collaboration requires mutual effort across institutions to work more closely together. In the case of other UK projects, by the midterm of the IHR Project, some of these other projects were still nascent.

The Itad team also found that the IHR Project could be more effective if the technical assistance activities were accompanied by relevant materials and equipment, such as laboratory equipment and reagents. In Nigeria, for example, these additional inputs had not been part of the original project business case as international procurement of resources in PHE IHR Project countries was not an area of expertise for PHE. However, there was subsequent recognition of the need for some limited capital investment to embed the changes brought about by the technical assistance offered and thus the project applied for and obtained a variation to its business case and MoU from DHSC to allow limited capital investment from existing funds.

While the Itad MTE team recommended that some funds be made available to complement the Project’s technical assistance, it was also found that the IHR Project could be more strategic in leveraging other UK Aid processes and initiatives as part of the ‘One HMG^11^’ harmonisation and complementarity that the UK government encourages all of its departments to employ in the countries where it is providing development assistance. As a technical agency of DHSC, PHE can only make recommendations on how to use funding, and adjustments on the basis of these recommendations were occasionally implemented. For instance, during the Nigeria meningitis outbreak in 2018, DFID Nigeria redirected additional funding to support WASH (water, sanitation and hygiene) activities as part of the outbreak response. PHE leads in other countries were also in discussion with DFID health and humanitarian teams about where health security funding for PHE partner institutions could be included in other DFID project business cases. Other opportunities that were noted included working with and through Foreign and Commonwealth Office (FCO) diplomatic networks to advocate for increased health security funding from host governments.

It was more difficult for the MTE team to assess the degree to which the IHR Project inputs and processes were relevant for achieving project outcomes. This was mainly due to project design, where the original IHR Project theory of change and logical framework did not provide sufficient explanation of the underlying assumptions or of the conditions that needed to be met in order for project inputs and activities to translate into outcomes and impact. This is covered in more detail below. The theory of change and logframe have since been updated as the project matures.

### Is the project doing things the right way?

The IHR Project was set up on adaptive programming principles, which meant being as flexible and responsive as possible, given that the national and sub-national teams that they were supporting would have to change plans if they were confronted with a public health emergency that required the NPHI to halt other activities and respond [7]. This meant that while annual work plans were developed with each country partner and with Africa CDC, they often could not be implemented as originally envisaged; either during the time frame agreed, or in some cases, not at all. The adaptive nature of the project has been brought into sharp relief in 2020 with the COVID-19 pandemic, as external face-to-face technical assistance has had to be reduced or dispensed with completely in most countries, though support has continued to be provided by in-country team members and through virtual platforms by specialists based in the UK.

PHE’s technical assistance approach is outlined in Table 3. Up to the point of the midterm evaluation, the IHR Project at country-level was structured around having an in-country based Country Lead, as well as an Africa CDC lead. Country Leads’ responsibilities included some direct technical public health support as well as building and maintaining good relations with the national public health institutions and other health security programmes in countries. These efforts were important for establishing partner priorities and needs and for coordinating with PHE technical specialists to determine, agree and support the implementation of a national annual work plan. The technical aspects of the work plan were then undertaken by PHE technical specialists who conduct short-term missions in-country – usually between one to two weeks. The exception to this is how the IHR Project operates in Pakistan, with two technical Public Health consultants and a larger complement of locally engaged technical staff. As such, most of the training and mentoring activities were undertaken by the in-country technical team with occasional support from PHE’s UK based teams.

**Table 3:** PHE’s model of Technical Assistance at the time of the MTE

• Primarily UK based, albeit with country-based technical staff (e.g. Country Leads, epidemiologist in Africa CDC)) sitting alongside partner institutions.	
• Short-term (i.e. based around a series of short visits of up to two weeks – except for the Pakistan model), although with longer-term posts being set up in all countries by end of 2019.	
• Supported by Country Lead.	
• Provision of training courses – sometimes in-country, sometimes international.	
• Seconded technical assistance.	
• Supporting field visits (i.e. regional or international).	

Key informant interviews with country stakeholders indicated that, while most had high regard for the expertise of the PHE technical specialists, they would have gained more if these individuals could spend more time in-country, working with their counterpart teams to consolidate the knowledge and skills that they were helping to develop. These concerns with ‘fly in-fly out’ type of technical assistance are well known [8, 9]. It was found that the Project had employed many of the features of what can make short-term technical assistance more effective, including trying to foster shared ownership of the capability development offered by the Project and using a peer-to-peer approach that has been well received by countries^12^. Anecdotal evidence was also found of where technical assistance in the form of training and follow-up mentoring had led to improvements in practice and in IHR compliance, but these were not being systematically captured by the Project’s routine reporting (see ‘Right results’ section below for further detail).

From our interviews it was clear that the PHE teams had found some initial challenges in building relationships with some country partners, partly because PHE is not a typical donor, in that it does not provide funds, but rather provides timely technical assistance, while national and regional stakeholders had expected to receive both technical assistance and funding. It is important to note here however from the perspective of doing things the ‘right way’, that project staff and management had mixed views on whether including funding for material and equipment would be appropriate, as many felt that if they did have a pot of funds that their relationship would change from one of advisory technical partnership to one that was more akin to donor and recipient.

The IHR Project management and implementation arrangements were found to be particularly complicated. PHE is a complex organisation being a relatively new entity, made up of what were more than 70 separate public health organisations prior to 2013. Each PHE division has its own working culture and management structures, including the PHE Global Public Health Directorate. PHE is an executive agency of the UK DHSC, operating under the terms of a framework agreement and has operated as a ‘distinct delivery organisation with operational autonomy’. As of this writing, PHE is currently undergoing another re-organisation as it is being replaced with a new organisation.^13^

Bringing together the considerable expertise found within different parts of the organisation to contribute to the IHR Project took additional effort. The IHR Project team was initially fairly centralised, with all approvals for funding and travel arrangements, for example, going through the senior management team based in London. Country Leads worked with their partners to develop annual work plans, which were consolidated into an overall IHR Project annual plan and budget. However, the Country Leads were not provided with a dedicated budget or delegated authority to adjust budgets as circumstances changed. This allowed the IHR Project senior management to exercise maximum flexibility across the entirety of the project, but sometimes reduced in-country responsiveness, as all changes had to be agreed by the London-based team.

The RACI analysis suggested that, initially, there was some confusion and overlap of responsibilities between individuals and groups. An example of this was the relative roles of the IHR Project Lead, technical team leads and Country Leads who are all jointly responsible/ accountable for a number of functions and processes related to the design, implementation, administration and oversight of the IHR Project. Key informant interviews further suggested that there had been a lack of clarity among some engaged stakeholders over what different teams are responsible and accountable for; how these responsibilities/accountabilities overlap with other teams; and how individual team inputs contribute to the overall goals and objectives of the IHR project.

The RACI analysis has also highlighted that while multiple stakeholders were often jointly responsible or accountable for project functions and processes, the IHR Project leadership team were ultimately accountable for overall project performance and retained significant control over key operational project functions and processes (e.g., budgeting, travel and spending approvals). Stakeholders noted that this heavy workload and the requirement for a limited number of people to sign off on most approvals/processes had at times created bottlenecks and delays which could have been avoided if more individuals had had sufficient authority to sign off. During the MTE period the PHE IHR Project re-organised the project leadership and related administration, to include a Project Lead for Africa, Project Lead for the Asia and a cross-cutting role on governance to help resolve some of these issues.

The IHR Project’s methods for monitoring project progress proved especially challenging from the perspective of the evaluation. The monitoring system being used was Jira, which is essentially a project task management tool where documents can be uploaded against a task and inputs are linked to project outputs and outcomes. Each deliverable on Jira is mapped to the logframe, creating a repository of evidence of delivery against logframe outputs and outcomes and associated JEE or State Party Self-Assessment and Reporting (SPAR) indicators [10]. However, at the time of the MTE, the monitoring process was still being developed and there was little reporting against outcome, JEE or SPAR indicators. This was partly due to the fact that there were considerable problems with the structure of the Project’s logical framework, and a weak relationship between the logframe and the Project’s original TOC. This was unfortunate as our country visits found that there were good examples of where the IHR Project was making progress towards what could be considered outcome-level targets, but this was not being adequately captured through the Project’s routine monitoring systems; this is explored further in the ‘getting the right results’ section below.

### Getting the right results

For the purposes of the evaluation, Itad found that the IHR Project needed to identify more explicit project outcomes and a clearer change pathway between the IHR Project interventions and these outcomes.

Although the project to date had not been able to articulate the connections across the results chain, from inputs and activities through to outcomes and impact, the MTE did find evidence that some project outputs were contributing to the achievement of the desired outcomes in some technical areas and in some countries. Table 4 provides examples of where progress against project-level outcomes as reflected in the TOC was being made at the time of the MTE.

**Table 4:** IHR Project progress on outcomes as reflected in the TOC

• There was some evidence of strengthened system coordination and collaboration through NPHIs in some countries. More limited progress has been made at the regional and global levels.	
• The IHR Project has made at least some contribution to improvements in health workforce capacity in some technical areas and in some countries.	
• The IHR Project has contributed to the strengthening and expansion of some public health technical systems in some countries.	
There was evidence of improving cross-government coordination for public health system strengthening.	

One of the project objectives was to strengthen system coordination in order to reduce potential fragmentation of emergency prevention, preparedness and response efforts. There are numerous cross-cutting themes around IHR related coordination in countries, including coordinating within a ‘One Health’ framework (including human, animal and environmental health) [11–14]; donor coordination (ensuring donor inputs are aligned with government priorities and are harmonised to maximise the effectiveness of donor funds) [15, 16]; and cross HMG coordination (including UK entities working in a country, such as PHE, DFID, the FCO, the Ministry of Defence amongst others). The project had begun to have some successes in helping NPHIs to improve the coordination of their IHR efforts, while also striving to be a constructive and active partner in One HMG discussions.

It was particularly difficult for the MTE team to find sufficient, objective data about how effective the IHR Project technical assistance had been in improving IHR capabilities, other than the opinions of stakeholders who were satisfied with the training they had received. It was also difficult to find evidence from IHR Project documents of how workshops, simulation exercises and one-to-one mentoring had led to improved practices of those who had attended the trainings. Anecdotal evidence suggested that the technical assistance had contributed to improved practice, as highlighted in Table 5.

**Table 5:** Indicative examples of where the PHE IHR Project technical assistance facilitated improved practices

• Two district teams trained by the IHR Project in Pakistan had successfully integrated disease surveillance into their routine data collection through DHIS2 and had facilitated 100% of health facilities to report on a weekly basis.	
• The IHR Project in Ethiopia has invested considerable technical support in training and mentoring the National Poison Centre team, who had then set up an emergency call centre and are using ToxBase to help hospitals across the country to diagnose and treat poisoning cases more quickly.	
• In Nigeria, the IHR Project has supported the Nigeria CDC team to enhance their emergency preparedness, resilience and response (EPRR) capacities, with evidence from periodic ‘Keep Pushing’ exercises indicated that EPRR practice has subsequently improved.	

## Discussion

We have identified the following key learning from the results presented above, which may be of value to other practitioners engaged in initiatives to strengthen IHR or GHS capacities in LMICs.

### Establishing effective relationships

Alari and Thomas (2016), in a review of efforts to improve the effectiveness of government institutions, found that one critical success factor included establishing “relationships and trust needed to create entry points for a project” [17]. One of the essential features of the PHE IHR project was the time taken by PHE teams to scope what IHR areas the project should prioritise and to develop relationships with key national government officials. By taking time to build these relationships, especially through PHE’s technical specialists, it’s apparent that PHE teams were able to create entry points for introducing capacity development interventions that are more likely to be effective.

The PHE project’s experience highlights potential ways to strengthen effective relationships, and reinforces lessons learned by others:

- *Build in adequate length for an inception period and early phases of the project* to develop relationships with key stakeholders across organisations, especially those that are in direct counterpart positions, ensuring that there is a mutual understanding of what the IHR are, what the country’s IHR compliance status is and what needs to be done to strengthen this compliance. This is especially important when it comes to health security as many countries are challenged with delivering routine services and often do not have the bandwidth to prioritise emergency preparedness and response mechanisms [18];
- *Offer technical support on a ‘peer-to-peer’ basis*. Whilst there is surprisingly little literature on the relative effectiveness of peer-to-peer technical support, the few evaluative reports that are available (mostly on south-south support) suggest that this can yield greater individual and institutional development.^14^ Experience from the PHE project suggests that this is strengthened if delivered by individuals with strong technical experience in the related IHR field, and who may still be providing similar support in their own country or other countries to lend credibility to their advice and support. The peer-to-peer support from one public health institute (PHE) to other national public health institutes appears to be especially appreciated and increases the potential to be effective.

### Establishing effective management and monitoring systems

The IHR Strengthening project was committed to applying adaptive management approaches. Intrac, in their own overview of adaptive management, suggest that adaptive management can be ‘tactical’ or ‘strategic’, whereby tactical adaptation means tweaking interventions in response to feedback or data and ‘strategic’ adaptation requires collection, analysis and use of more comprehensive data to examine progress towards project outcomes and to foster a learning culture [19]. Other authors [20–22] reinforce the need for data-driven learning and adaptation based on their own studies. Although the IHR Project management team highlighted the importance of being adaptive in how they work in each country’s context, Itad found that most adaptation was driven by specific circumstances and more tactical in nature but did not find much evidence of strategic-level adaptation, as it was not apparent how the project was using project output and outcome data to adjust its overall approach. The key lessons learned from this examination suggest that it is important to employ an adaptive management approach, underpinned by a theory of change and appropriate results framework (ie one that tracks progress across all levels of the theory of change), with the understanding that health security strengthening projects need to be, by their nature, complex, systems-oriented and subject to frequent adjustments to plans.

It is important to have a clear definition of the indicators in the results framework including clear plans for how they would be measured through routine reporting systems to track project progress. Itad found that the Joint External Evaluation (JEE) tool is one way to measure the system-level change or outcomes that the project is ultimately contributing towards, but the JEE is not conducted on a regular basis as it is fairly cumbersome. The PHE team noted that there are other tools that could also be used to measure this system-level outcomes, including the ‘State Party Self-Assessment and Reporting’ (SPAR) tool^15^, which is usually completed on an annual basis and has a high degree of overlap with the JEE framework [23]. At the time of the mid-term review, there hadn’t been a concerted effort to use SPAR results to map the IHR Project progress in the PHE project countries, or to use this for making project adjustments. A key lesson out of the PHE’s experience suggests that projects need to give sufficient attention to defining clear indicators, backed by quality data sources and monitoring tools (e.g., SPAR).

## Conclusions

The support that the IHR Project has provided to countries has never been needed more, as has been highlighted by the COVID-19 pandemic. All evidence gathered and analysed for the midterm evaluation indicated that the PHE IHR Project is highly relevant and valued by national counterparts and other health security stakeholders across the six countries and by the Africa CDC. Prior to the start of the IHR Project, PHE had experience of providing short-term technical and material support in response to major disease outbreaks, such as Ebola in West Africa, with limited prior experience of working on a longer-term basis in lower income countries. Nevertheless, PHE managed to set up a complex, longer-term project that has real potential to make a difference in a fairly short space of time.

At the midterm point of the project, there were clearly areas that could be enhanced especially in terms of having the means to track progress towards achieving project outcomes and paying more attention to how the capacities developed in countries are likely to be sustained. Despite this, there was good evidence that the project was doing the right things and aligning its work in the right way, with more limited evidence at the time of the midterm evaluation that it was making progress towards achieving the right results. An endline evaluation took place in 2021 and the report is still under development.

## Data Availability

Data sharing is not applicable to this article as no quantitative datasets were generated or analysed during the current study.

## Abbreviations

ACDC: Africa Centres for Disease Control
AFRO: Africa Regional Offices
CDC: Centres for Disease Control
DFID: Department for International Development
DHSC: Department of Health and Social Care
GHS: Global Health Security
IHR: International Health Regulations
JEE: Joint External Evaluation
M&E: Monitoring and Evaluation
MTE: Midterm Evaluation
NPCC: National Poisons Control Centre (Myanmar)
NPHI: National Public Health Institute
ODA: Official Development Assistance
PHE: Public Health England
SPAR: State Party Self-Assessment and Reporting
TPM&E: Third-Party Monitoring And Evaluation
UK: United Kingdom
WHO: World Health Organisation

## Footnotes

1 Referred to as the ‘IHR Project’ or ‘the Project’ from this point forward.

2 Or in the case of regional organisations such as Africa CDC, support their Member states to implement their national IHR commitments.

3 International GHS literature included World Health Organisation best practice and IHR related guidance documents; US Centres for Disease Control and Prevention GHS guidance and progress reports; progress reports on regional GHS responses, and academic articles on adaptive management.

4 Country-level reports included health sector strategies, health emergency action plans, reports of national epidemic responses (e.g., on Ebola in Sierra Leone, and SARS in Pakistan).

5 A theory of change (TOC) provides an overview of how project inputs and interventions are meant to contribute to the desired change or results. The TOC should also describe the project’s assumptions that underpin making this contribution. (see https://www.betterevaluation.org/en/node/5280)

6 See John Mayne [24] (2011)

7 See https://thedigitalprojectmanager.com/raci-chart-made-simple// for further information.

8 See https://wmich.edu/sites/default/files/attachments/u350/2014/UFE_checklist_2013.pdf for a detailed explanation of utilisation-focused evaluations.

9 As a signatory to the IHR, countries may voluntarily undergo a Joint External Evaluation to identify areas where countries are performing well and where they need to improve their disease and risk prevention, surveillance, response and control measures. See footnote 10 above and https://www.who.int/health-topics/international-health-regulations#tab=tab_1 for further information.

10 Such as the DHSC funded UK Public Health Rapid Support Team programme and Fleming Fund grants.

11 ‘HMG’ refers to ‘Her Majesty’s Government’. ‘One HMG’ targets ensuring more efficient and joined up use of UK government resources across different programmes within countries.

12 Ibid

13 A recent announcement (August 2020) by the Secretariat State for Health and Social Care indicated that PHE will be abolished and partially replaced by a new National Institute for Health Protection.

14 ibid.

15 SPAR is the IHR Self-Assessment Annual Reporting Tool was released by WHO in 2018. https://extranet.who.int/sph/news/ihr-self-assessment-annual-reporting-tool-spar-2018
